# Three‐Dimensional Analysis of Maxillary Incisor Root Resorption Associated With Three Distinct Anterior Retraction Protocols: A Retrospective Study

**DOI:** 10.1155/ijod/5583614

**Published:** 2026-02-26

**Authors:** Huimin Cheng, Yuhao Huang, Yanjun Pan, Tianhui He, Hong Ai, Zhihui Mai

**Affiliations:** ^1^ Department of Stomatology, The Third Affiliated Hospital of Sun Yat-Sen University, Guangzhou, Guang Dong, China, zssy.com.cn; ^2^ Department of Stomatology, Shunde Women and Children’s Hospital of Guang Dong Medical University (Maternal and Child Healthcare Hospital of ShunDe Foshan), Foshan, Guang Dong, China

**Keywords:** CBCT, EARR, fixed aligner: microimplant support, invisible aligner

## Abstract

**Objective:**

Our objective was to compare differences in the amount of apical external resorption of maxillary incisor roots before and after orthodontic treatment with an invisible aligner, fixed orthodontic without microimplant support, and fixed orthodontic with microimplant support.

**Methods:**

This study collected the clinical data of patients with Class II malocclusion who underwent orthodontic treatment with the treatment plan of extracting four premolars between 2018 and 2022. There were 10 patients treated with an invisible aligner, 10 treated with a fixed orthodontic without microimplant support group, and 10 treated with a fixed orthodontic with microimplant support. Changes in root volume and the length of the maxillary incisors in the three groups before and after the treatments were measured using cone beam computed tomography (CBCT) and 3D‐Slicer software.

**Results:**

The *t*‐test results showed that after treatment, the root volume and length of maxillary incisors changed in all three groups of patients. The differences in volume and length before and after treatment within each group were statistically significant, but there were no statistically significant differences in the changes between the three groups. The amount of change of the root volume of the maxillary central incisors was significantly greater than that of the maxillary lateral incisors. Correlation analysis showed that the amount of change in the length of the roots was positively correlated with age.

**Conclusion:**

Fixed orthodontic treatment with microimplant support had a greater effect on the root volume of the maxillary incisors than invisible orthodontic treatment and conventional fixed orthodontic treatment. The degree of external apical root resorption (EARR) was positively correlated with the patient’s age. Therefore, in clinical practice, for older patients or those requiring the use of mini‐implants for retraction, special attention should be paid to the root resorption.

## 1. Introduction

Orthodontically‐induced root resorption (external apical root resorption [EARR]), which typically occurs in the apical region of the root [1], is caused by various mechanical pressures and is one of the most common complications of orthodontic treatment [2, 3]. The causes of root resorption in orthodontics remain unclear, though Lopatiene and Dumbravaite [4] categorized contributing factors into biological and mechanical groups. Biological factors include genetics, nutrition, diseases, medications, hormones, age, gender, root morphology, root development, erupted teeth, alveolar bone density, dental trauma, root canal treatment, type of malocclusion, tooth position, and vertical bone contour [4]. Mechanical factors include appliance type, method and distance of tooth movement, force magnitude, treatment duration, and tooth extraction [5]. Higher force, longer movement distance, and increased tooth depression are associated with greater root resorption [6].

In Class II patients who underwent extraction of four premolars for orthodontic purposes, a large amount of retraction of the upper anterior teeth is required, which increase the chance and degree of root resorption, particularly in skeletal Class II malocclusion patients who often require maxillary implant support, where torque control and depression of the upper anterior teeth further exacerbate the risk [7]. Fixed orthodontic treatment of the upper anterior teeth applies continuous force, mainly concentrated at the root apex, which increase both the pressure and the risk of root resorption [7, 8]. Although the application of microimplants has enhanced esthetics, convenience, and comfort for patients, it may also increase the risk of EARR [9].

Invisible aligners, also known as clear aligners, are removable orthodontic devices designed to straighten teeth using controlled, minimal intermittent force [10]. In recent years, invisible appliances have been used in extraction cases. However, their impact on EARR remains underresearched. The study pointed out that Invisalign can generate large transient stresses during repeated daily removal, which may lead to loosening of teeth and pathologic resorption of the alveolar bone [11, 12].

Cone beam computed tomography (CBCT) has 89% reliability in diagnosing EARR and is a reliable tool for detecting extra‐apical resorption [13, 14]. In previous studies, conventional 2‐dimensional images have been used to measure the root length before and after treatment, but have neglected to assess the amount of change in root volume. Root volume measurements based on CBCT reconstructions can complement the limitations of root length measurements alone, and the combination of volume and length changes can be considered as an indicator when assessing the level of root resorption to improve the sensitivity and accuracy of clinical diagnosis [15].

Therefore, the purpose of this study is to evaluate the length and volume changes of maxillary incisors before and after orthodontic treatment using CBCT and 3D Slicer software, and to analyze the differences in different treatment methods for patients treated with invisible orthodontic appliances, fixed orthodontic treatment without microimplant support, and fixed orthodontic treatment with micro implant support [16].

## 2. Material and Methods

### 2.1. Patients

The records of Class II patients who underwent extraction of four premolars for orthodontic purposes and were treated from 2018 to 2022 were reviewed. The study was approved by the Ethics Committee (II2024‐197‐01).

Inclusion criteria were: (1) Age ≥ 18 years; (2) ANB ≥ 4°; (3) mild crowding; (4) anterior Class II maxillary protrusion; (5) orthodontic treatment plan was extraction of four first premolar teeth, designed with strong support; (6) good periodontal condition and no history of periodontal disease; (7) no apical lesions or root canal treatment; (8) no prior orthodontic treatment; (9) no temporomandibular joint disease or obvious abnormality of bilateral condyles; (10) data of interest were complete and the patient received CBCT before and after treatment and images were available; (11) no incisor root fractures.

Based on the above criteria, 30 patients (total of 120 maxillary incisors) were included in the study. There were nine males and 21 females; the mean patient age was 24 ± 6 years; the mean treatment duration was 37 ± 11 months.

### 2.2. Grouping

1. Invisible aligner group: Patients were treated with Invisalign invisible aligners (USA), which required wearing the aligners daily for ≥22 h. During the retraction phase, the anterior teeth are retracted by approximately 0.2 mm per aligner. Replace an aligner every 7 days on average. The aligners resulted in a Class I occlusal relation between the cusp and molar teeth, with the anterior teeth overlapped and covered by approximately 1 mm.
2. Fixed orthodontic without microimplant support group: Patients were treated with metal self‐ligating aligners (DamonQ; Ormco; USA). Nickel titanium wires were used for the initial alignment and leveling phases, with sizes of 0.012, 0.014, 0.016, and 0.018‐inch. For closing the extraction gap stainless steel wires of 0.017 inches × 0.022 inches, 0.019 inches × 0.025 inches were used. Connect the posterior tooth bracket traction hook to the short traction hook on the archwire, and replace the elastic rubber band every 4 weeks to close the gap until the orthodontic treatment is completed.
3. Fixed orthodontic with microimplant support group: Patients were treated the same as that of fixed group; however, microimplants (Dentos; 0.8 mm × 10 mm; South Korea) were implanted between the maxillary first molar and second molar, at a height of about 8 mm from the attached gingiva. The microimplants were placed immediately after the operation, and elastic rubber bands were used to apply a force of about 70 g on each side of the microimplant. The microimplants were connected to the short traction hooks on the archwire, and the elastic rubber bands were replaced every 4 weeks to close the gap until the end of the orthodontic treatment.

### 2.3. Imaging Measurements

Images of the patients before and after treatment were obtained with CBCT and the NNT Viewer (software version 11.5; Italy). All CBCTs were performed by the same practitioner, after which the results were imported in DICOM format into 3D‐Slicer software (version 5.7.0), and the maxillary incisor root length and volume were measured.

#### 2.3.1. Root Volume Measurement and Calculation

Sagittal, coronal, and axial plan images were used for threshold segmentation preprocessing of the anatomical morphology of the maxillary incisors, and the segmentation results were used to create a mask. Precise segmentation of the maxillary incisors was performed based on differences of the gray values of the images on the newly created mask. Based on the gray value differences between the enamel, osteoid, and alveolar bone images, frame‐by‐frame deletion, supplementation, and modification were performed in the sagittal, coronal, and axial planes to obtain an accurate 3D model of the maxillary incisors (Figure 1). The roots of the incisors were segmented based on the enamel–osteoplastic boundaries (Figure 2). The root volume of each incisor was measured using 3D‐Slicer software version 5.7.0, and averaged over three measurements.

Figure 1(A) Divide the roots of maxillary incisors. (B) Divide the crowns and roots along the enamel–osteoskeletal boundary. (C) Separate the roots under the alveolar bone.

**Figure:**
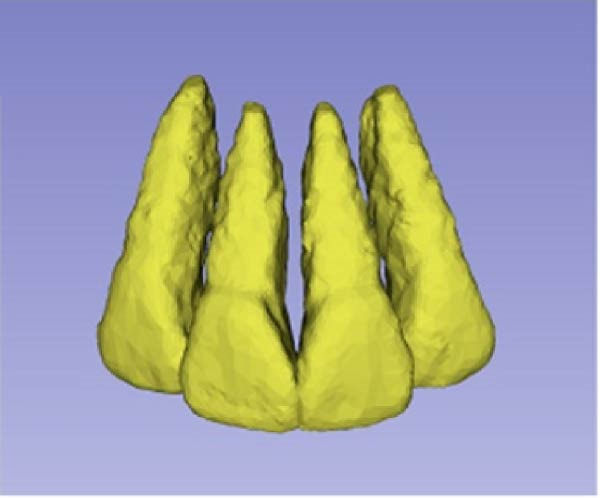
(A)

**Figure:**
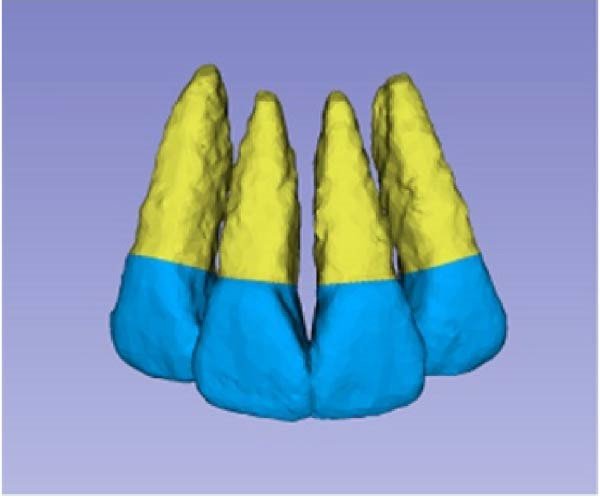
(B)

**Figure:**
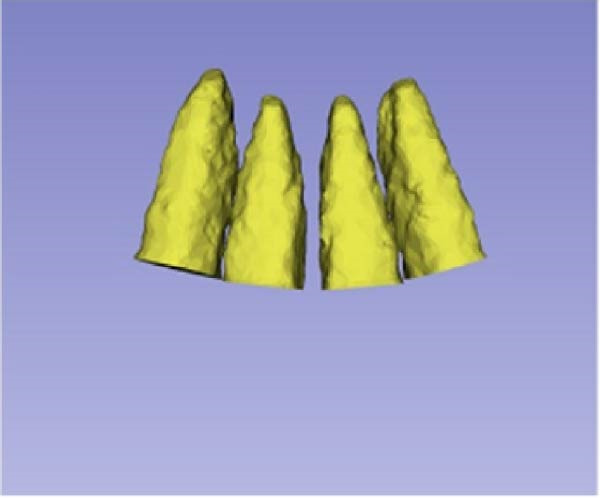
(C)

Figure 2(A) Axial surface of the maxillary incisor. (B) Straight line distance between the center division point of the root neck and the apical point of the transenamel osteoid boundary was defined as the root length. (C) Coronal surface of the maxillary incisor. (D) Sagittal surface of the maxillary incisor.

**Figure:**
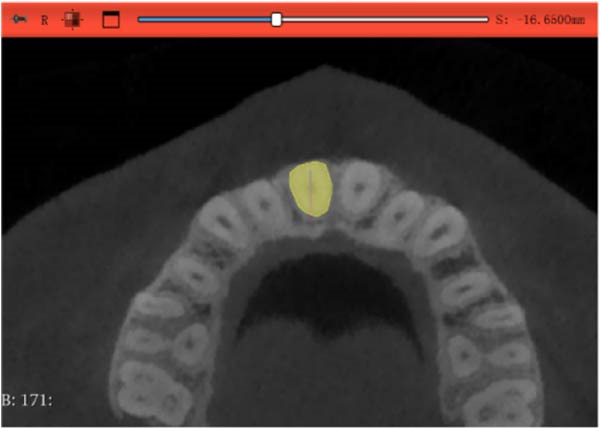
(A)

**Figure:**
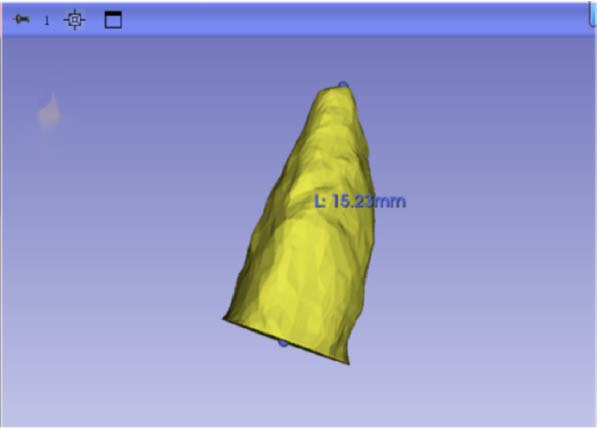
(B)

**Figure:**
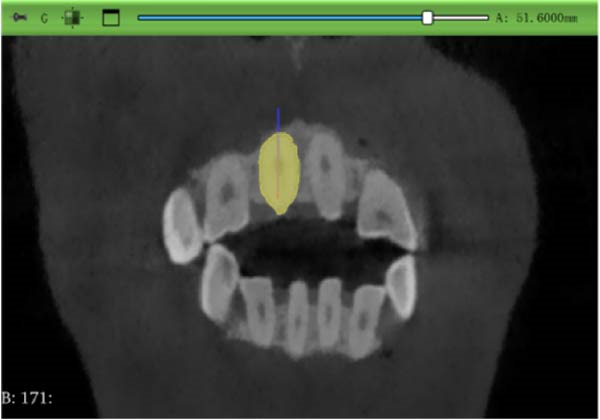
(C)

**Figure:**
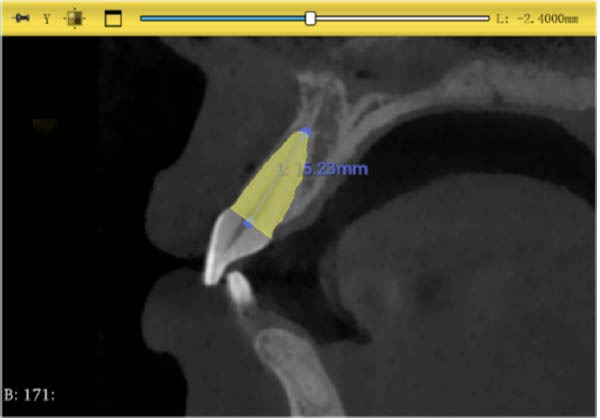
(D)

#### 2.3.2. Root Length Measurement

The center point of the root neck and the apical point of the incisors through the enamel–osteum boundary were determined on the median sagittal plane image of the maxillary incisors. The straight‐line distance between the two points was defined as the root length, and this line was visible in the 3D template through the root canal of the tooth (Figure 3).

Figure 3Changes in incisor volume and length before and after treatment. (A) Volume of upper‐central incisor (mm^3^). (B) Length of central incisor (mm). (C) Volume of lateral incisor (mm^3^). (D) Length of upper‐lateral incisor (mm).  ^∗∗∗^ *p* < 0.001;  ^∗∗∗^ *p* < 0.0001.

**Figure:**
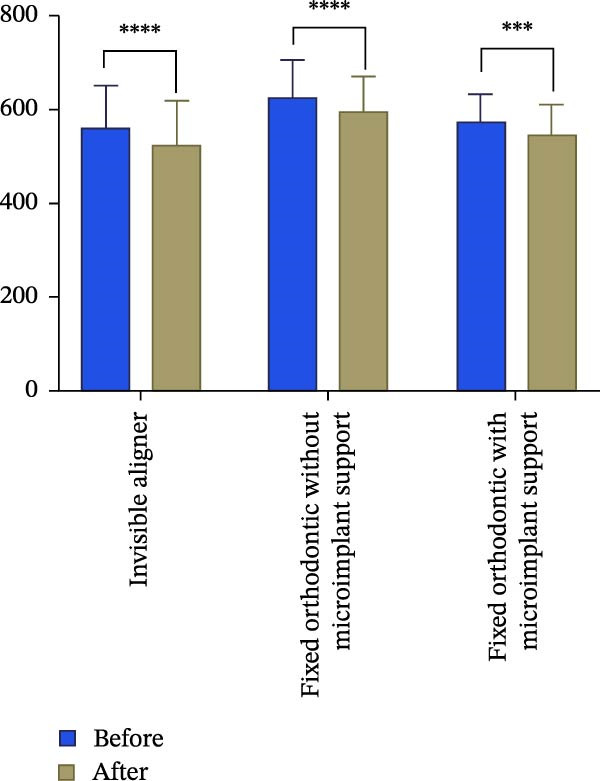
(A)

**Figure:**
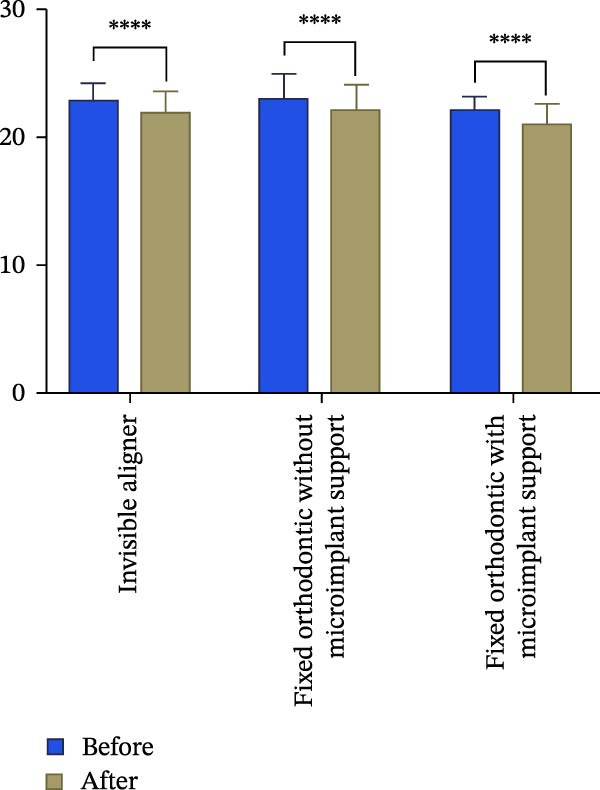
(B)

**Figure:**
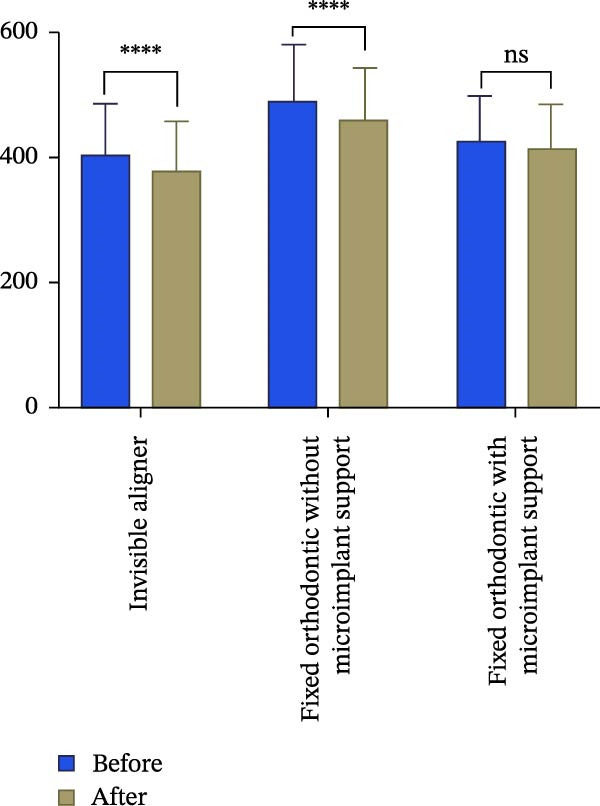
(C)

**Figure:**
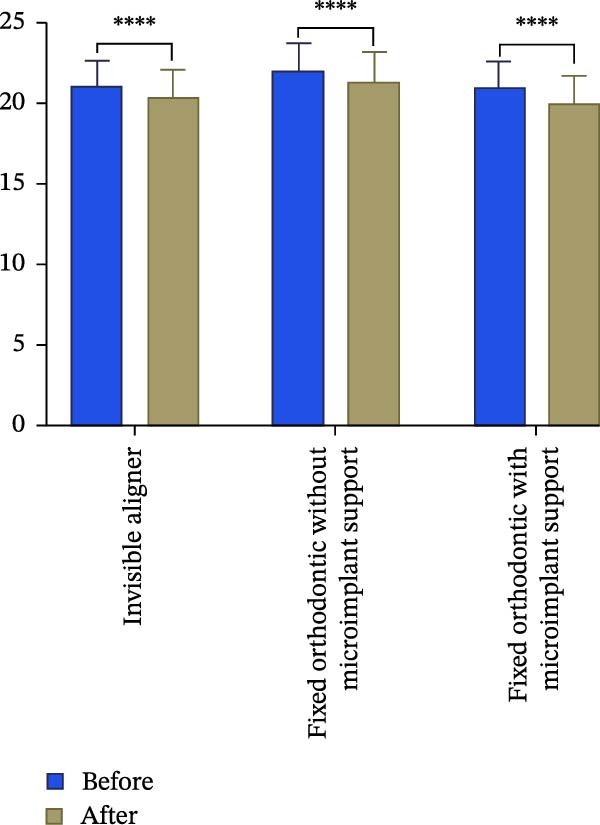
(D)

#### 2.3.3. Calculations

Changes in root volume and length were calculated with the following formulas:

Amount of change in root volume=Pretreatment root volume−posttreatment root volume.

Amount of change in root length=Pretreatment root length−posttreatment root length.

Percentage change in volume=Pretreatment root volume−posttreatment root volume/pretreatment root volume ×100%.

A volume change of ≤10% was considered mild resorption; a volume changed of 10%–20% was considered moderate resorption; a change >20% was considered severe resorption.

## 3. Statistical Analysis

The Shapiro–Wilk test was used to assess the normality of the distribution. Changes in root length and volume before and after treatment were compared using either the *t*‐test or Mann–Whitney test. The correlation between changes in length and volume, age, and treatment duration was analyzed using Pearson correlation analysis, while the correlation with gender was analyzed using Spearman correlation analysis.

All statistical analyses were performed using SPSS software (version 20.0; IBM, Armonk, NY). A *p*‐value of <0.05 was considered statistically significant.

## 4. Results

### 4.1. Relations Between Treatment Methods and Changes in Root Length and Volume

Changes in incisal root length were seen in all three groups after treatment, and differences from the pretreatment values were statistically significant (*p*  < 0.05; Figure 3B,D). The posttreatment volume of the central incisors was also statistically different from the pretreatment values in all three groups (*p*  < 0.001). The posttreatment and pretreatment volume of the lateral incisors was significantly different only in groups invisible aligner and fixed orthodontic without microimplant support (Figure 3A,B). A two‐by‐two comparison showed that the amount of change in the volume of central incisors in fixed orthodontic with microimplant support group was greater than that in other two groups with statistically significant differences, but there was no statistically significant difference in the amount of change in the volume and length of lateral incisors among the three groups (Figure 4). The amount of volume change percentage resorption was 80% in the 10% category, 11% in the 10%–20% category, and 0% in the >20% category.

Figure 4Comparison of volume and length changes of incisors between three methods. (A) Comparison of volume changes of central incisors in the three groups. (B) Comparison of length changes of central incisors in the three groups. (C) Comparison of volume changes of lateral incisors in the three groups. (D) Comparison of length changes of lateral incisors in the three groups.  ^∗∗∗^ *p* < 0.0001.

**Figure:**
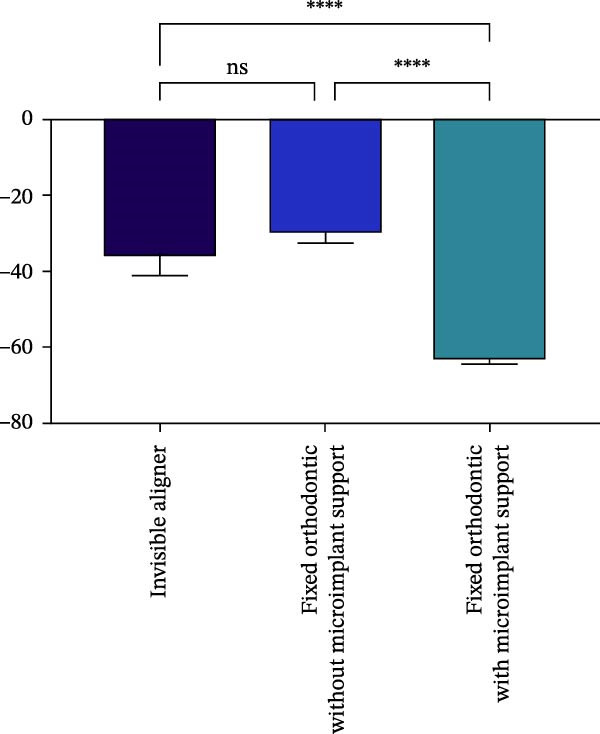
(A)

**Figure:**
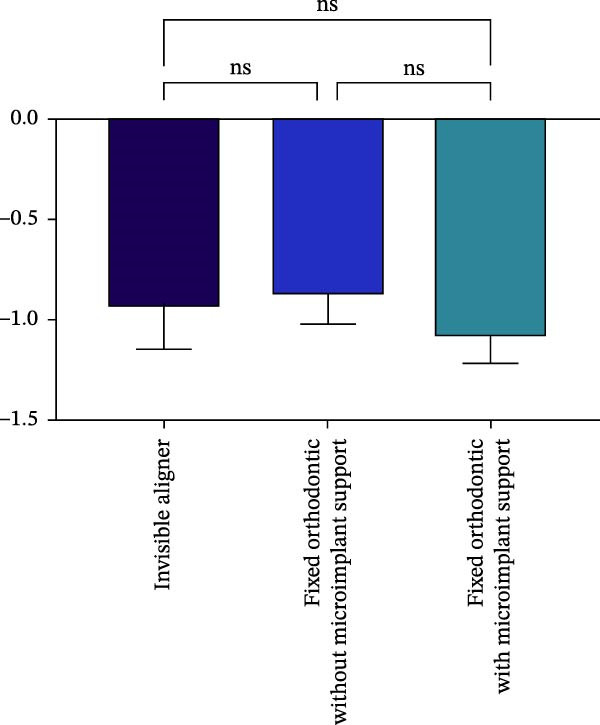
(B)

**Figure:**
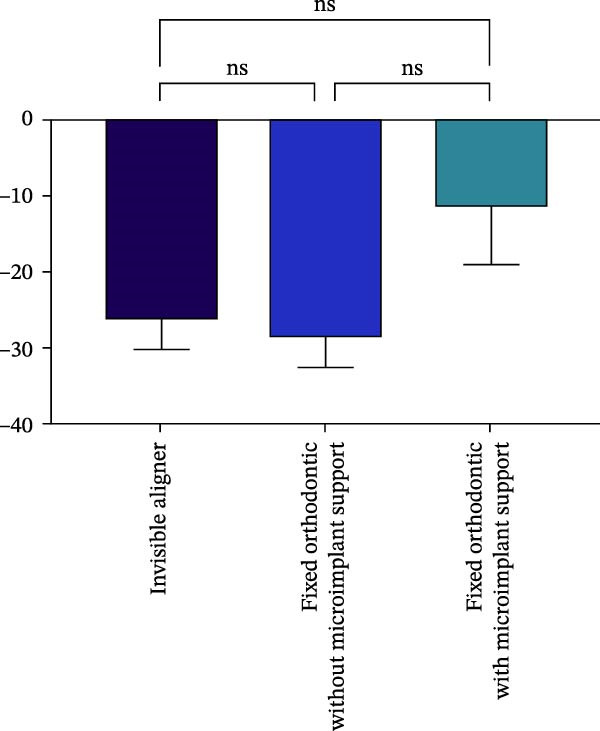
(C)

**Figure:**
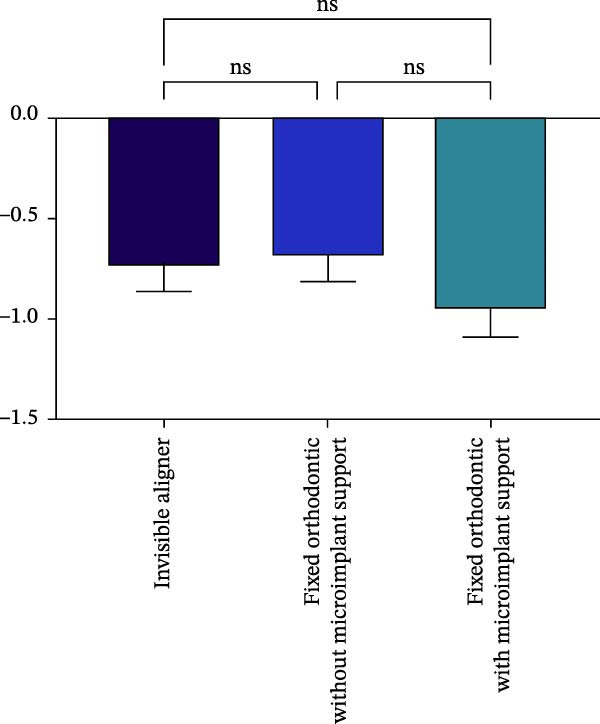
(D)

### 4.2. Relations Between Tooth Position and Root Resorption

Analysis of the volume changes of the central and lateral incisors showed that there was no statistically significant change in the volume and length of the central and lateral incisors before and after treatment (Figure 5).

Figure 5Comparison of length (A) and volume (B) changes between central and lateral incisors in three methods.

**Figure:**
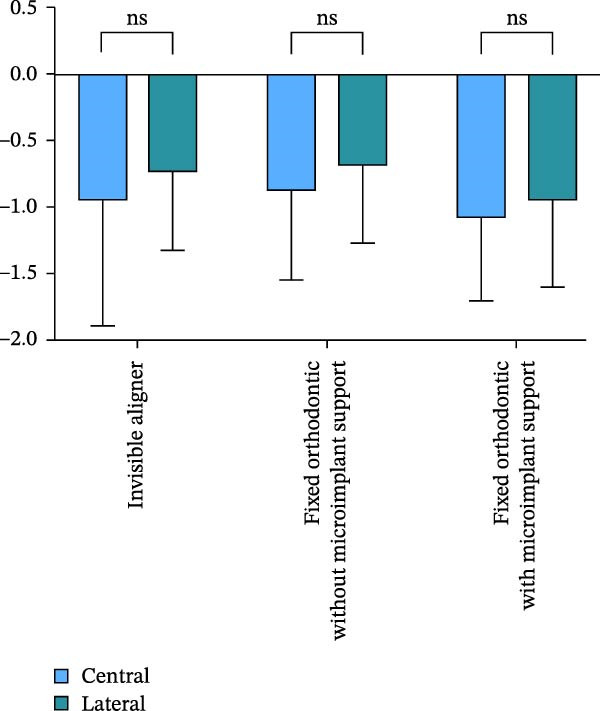
(A)

**Figure:**
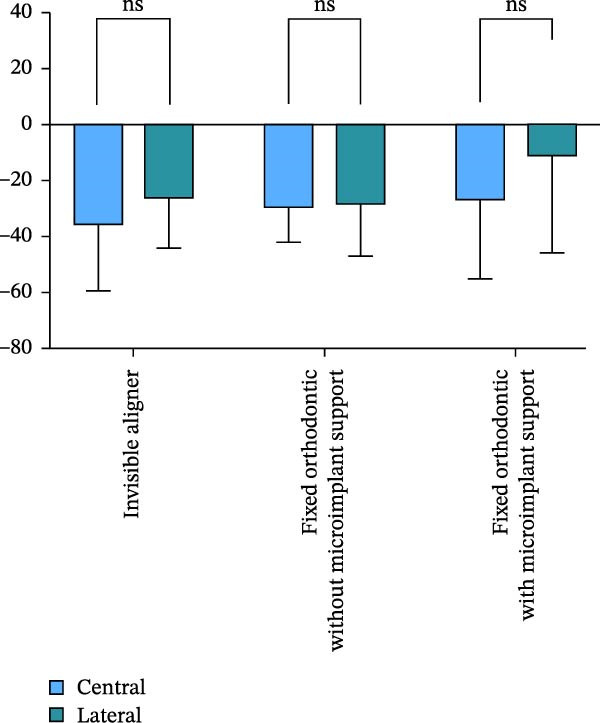
(B)

### 4.3. Relations Between Root Resorption and Sex, Age, and Treatment Duration

Pearson’s correlation analysis showed a moderate positive correlation between the amount of change in mesial incisor length before and after treatment and age with a correlation value of 0.4013 (*p*  < 0.05), whereas the correlation between the change in volume of central incisors, the change in volume and length of lateral incisors, and age and the treatment period was not significant. Spearman’s correlation analysis showed that the change in length of incisors before and after treatment did not have a significant correlation with gender (Table 1).

**Table 1:** The correlation analysis results of changes in the length and volume of the incisors with age, gender, and treatment duration.

Pearson’s correlation analysis	Volume of central incisors	Length of central incisors	Volume of lateral incisors	Length of lateral incisors
Age	0.1614	0.4013 ^∗^	0.02329	0.3431
Duration of treatment	0.009813	−0.1279	−0.02126	−0.2927
Sex	0.00525	−0.06616	−0.1281	−0.08611

^∗^ *p* < 0.05.

## 5. Discussion

EARR is a common problem in orthodontic treatment, and severe EARR can lead to root shortening and bone thinning, which may cause tooth sensitivity, pain, and even loosening and loss [17, 18]. Therefore, finding the optimal orthodontic force values and appliances to achieve efficient tooth movement results and reduce the incidence of orthodontic complications is an important goal of orthodontic research. Invisible orthodontic treatment, traditional fixed orthodontic treatment, and fixed orthodontic treatment supplemented with microimplants have different mechanical characteristics, and it is important to study the relation between EARR and these three methods.

To monitor EARR, various methods such as apical radiographs, surface tomography, and CBCT are commonly used. However, changes in crown torque before and after treatment can affect root length measurements obtained from 2D images, as these images are influenced by tooth inclination. Moreover, the palatal side of the root may experience resorption due to compression when the tooth is retracted inward. This makes it difficult to assess root volume changes with 2D radiographic techniques. In this study, we used CBCT to analyze the root length of upper incisors, aiming to minimize the impact of 2D image distortions and tooth inclination on resorption measurements. Additionally, volume measurements were performed to better assess overall root changes.

Deng et al. [14] pointed out that compared with the rigid and continuous orthodontic force of self‐ligating brackets, the invisible aligners deliver lighter and intermittent force, which gradually decrease as the appliance is worn overtime. Initially, friction between the aligners and attachments is high and substantial force is exerted on the tooth’s surface. However, as tooth movement progresses, the force diminishes, allowing for bone restoration and reducing the likelihood of root resorption [19]. In this study, we examined upper incisors in patients treated with invisible aligners, fixed braces, and fixed braces with microimplant support. Root resorption occurred in all three groups, but the length of resorption was similar across the groups. This suggests that although these three orthodontic methods differ mechanically, the direction and magnitude of the force can be controlled to minimize impact on root length during tooth movement.

Microimplants have the advantages of small size, high efficiency, no dependence on patient compliance, and protection of molar support [20]. A report showed that a patient in which microimplants were used to move the maxillary incisors with a lot of inward and downward movement, experienced severe and moderate root resorption in the maxillary central incisors and lateral incisors, respectively [21]. The results of this study showed that the effect of fixed orthodontic treatment with microimplant abutment on root length was not significantly different from that of the other two groups, but the effect on root volume was significantly greater than that of the other two groups. This may be attributed to the fact that the line of force of the anterior teeth that were internally retracted after placing the microimplant was closer to the center of the resistance, which resulted in the anterior teeth being subjected to greater force for overall movement, and the palatal side of the root was, therefore, subjected to greater pressure and resorption occurred. It has been shown that implant impedance is often accompanied by a depressive force when the anterior teeth are internally retracted, and this composite movement can cause more root resorption than simple anterior tooth retraction [22]. Therefore, using microimplants as support for anterior tooth adduction has certain reliability in clinical practice. However, it is still important to be cautious that implant support often comes with a downward force when retracting the front teeth. This comprehensive movement can cause more root resorption than simple anterior tooth retraction.

As invisible orthodontic technology and materials continue to evolve, their clinical applications are expanding. However, there remains debate about whether invisible orthodontic treatment can reduce root resorption [15, 23]. Fang et al. [19] found through meta‐analysis that invisible aligners could not completely avoid root resorption, but they did reduce both the incidence and severity of resorption [24]. Conversely, some researchers have found no significant difference in root resorption between invisible aligners and traditional fixed braces, suggesting that the choice of orthodontic appliance should not be based solely on the risk of root resorption [25]. Ke et al. [26] proposed that invisible aligners might better protect the roots of anterior teeth compared to fixed braces. Additionally, the removable nature of invisible aligners allows for intermittent force application, which provides recovery time for root tissues, thus, reducing the risk of resorption. While invisible aligners exert a large initial force, this force decays exponentially with wear time, which is less likely to cause root resorption compared to the sustained force of fixed aligners.

It has been reported that lateral incisors are more prone to EARR than central incisors [27, 28]. In the present study, the volume reduction of the root of the central incisor was higher than that of the lateral incisor before and after orthodontic treatment, although there was no significant difference in the volume change. This may be because the torque of the central incisors in Class II patients is relatively high and the roots of the central incisors are thicker. The alveolar bone in this area is thinner, making it easier to touch the cortical bone during movement. This result further suggests that in clinical practice, torque control should be included in risk considerations when retracting anterior teeth. Orthodontists should not only pay attention to the distance from the root apex to the cortical bone. At the same time, it is important to ensure that the middle and upper segments of the tooth root are always controlled within the trabecular bone of the alveolar bone, in order to avoid absorption caused by improper positioning of the tooth root.

Our correlation analysis revealed that the reduction in root length was positively correlated with age, and this result is consistent with previous findings by Nanekrungsan et al. [29], who observed an increased incidence of EARR with age. This may be due to the thinning of the periodontal membrane with age. Gandhi et al. [30] and Iglesias‐Linares et al. [31] concluded that the risk of EARR after orthodontic treatment is lower in adolescents than in adults because their roots are still developing, some of the apical foramina are not yet closed, and they have abundant blood flow. Costopoulos and Nanda [32] stated that root canal treated teeth are also less prone to EARR, which may be due to the increased mineral density and hardness after root canal treatment, resulting in decreased sensitivity to orthodontic forces.

Several other factors may influence the risk of EARR in Class II patients undergoing anterior tooth retraction. These include the amount of 3D directional movement, a history of root canal treatment, jawbone density, and root morphology. Specifically, patients with thick roots and thin alveolar bone have a reduced safe range for tooth movement. This highlights the importance of torque control when retracting anterior teeth. It is not only the distance from the apical region to the cortical bone that matters, but also ensuring that the root remains contained within the cancellous bone of the alveolar bone [24]. Understanding the mechanics of tooth movement and carefully monitoring treatment can prevent root resorption caused by improper force control [1].

A primary limitation of this study is the relatively small sample size. Further research with a larger cohort of patients is necessary to confirm these findings.

In conclusion, CBCT is reliable for diagnosing EARR, and also helps to assess alveolar bone limits, anterior root characteristics, and relations with surrounding anatomical structures [33]. Invisible orthodontics, fixed orthodontics, and fixed orthodontics with microimplants all cause a certain amount of root resorption, but there is no significant difference in the amount of resorption with appropriate force control. However, implant abutment may cause more resorption to occur on the palatal side of the root, resulting in a reduction in root volume. Choosing the appropriate method for a patient should take into account the patient characteristics, the biomechanical properties of the tooth root, the facts of tooth movement, and risk factors for root resorption.

## 6. Conclusion

Changes in the length and volume of incisors were observed with all three methods. Fixed orthodontic treatment with microimplant support had a greater effect on the root volume of the maxillary incisors than invisible orthodontic treatment and conventional fixed orthodontic treatment. The degree of EARR was positively correlated with the patient’s age. Therefore, in clinical practice, for older patients or those requiring the use of mini‐implants for retraction, special attention should be paid to the root resorption.

## Author Contributions

**Huimin Cheng:** data curation, writing – original draft preparation. **Yuhao Huang:** conceptualization, methodology, writing – original draft preparation. **Yanjun Pan:** formal analysis, software. **Tianhui He:** visualization, investigation. **Hong Ai**: supervision, validation. **Zhihui Mai:** writing – reviewing and editing, project administration.

## Funding

The authors received no funding for this work.

## Ethics Statement

The study was conducted according to the guidelines of the Declaration of Helsinki and approved by the Medical Ethics Committee of the Third Affiliated Hospital of Sun Yat‐sen University (Number II2024‐197‐01).

## Consent

The experiment was a retrospective experiment, and all enrolled patients had signed the informed consent form, and the clinical data could be used for teaching and scientific research.

## Conflicts of Interest

The authors declare no conflicts of interest.

## Data Availability

The datasets generated during and analyzed during the current study are available from the corresponding author upon reasonable request.
